# Safe Milk

**Published:** 1920-03-27

**Authors:** 


### SAFE MILK.

THERE is nowadays no great difficulty in furnishing bacteriologically "clean" milk to the inhabitants of cities and larger towns, the problems involved having gradually received a sympathetic hearing from local authorities and tradesmen alike but, as with all such organised advantages, they have been relatively poorly extended to the smaller communities. This is undoubtedly due to economic limitations. The amount of milk consumed is frequently considered too small to justify the installation expense, or the size of the population does not suggest that sufficient financial return can be expected. But in applying any method of sanitary control on the part of either the town or the milk-producer, it is well to remember that both the amount of milk consumed and the number of possible consumers largely determines the urgency of the local milk problem and the liability for disease to be started in the locality.

There are two well-known methods of obtaining safe milk, namely, (1) the addition of sanitary supervision of products and distribution; and (2) pasteurisation. Ideally we should like to see both processes generally employed, but in practice we must be satisfied with perfecting one of them to start with. The first, if carried to the point of "certified milk," will give an excellent degree of safety, but if trouble and expense are for any reason spared there is great liability that a sense of dangerous and false security may arise. The essential expert scientific supervision is impossible in remoter rural districts, and it is plain that this type of sanitary control will never be applicable to the smaller towns.

On the other hand, pasteurisation offers considerably more hope. It is stated that if a producer, or combination of producers, handle as much as 300 to 400 quarts of milk a day, a not unlikely amount, pasteurisation need not add more than a farthing or so to the cost per quart. A small pasteurising plant does not require an initial outlay beyond the means of the average small town, and in the experimental note from which our facts are taken, it is convincingly proved that a safe milk supply can be reasonably obtained by such means for any community consuming 400 quarts a day. The obvious difficulty is to persuade the rural producer himself to establish such development, and the only solution to the problem lies in a trial of municipal establishment of pasteurising plants. It is suggested that a local regulation be made requiring all milk sold to be first pasteurised. This pasteurisation should be done in a central plant owned and operated by the town and placed in conjunction with one of the various sources of steam power, which the majority of small townships nowadays possess. In the small type of plant it is suggested that milk should pass through the process after having been bottled, so that much of the opportunity for later contamination may be avoided. Also, in a small place, the arrangement enables a person at will to obtain the milk from his own animals or from his favourite dairyman.

We are aware that pasteurisation as a process has its known limitations, and that there are some who criticise the value of the reduction of bacterial content which it provides, but the fact remains that it is, all conditions considered, an invaluable means of checking the dissemination of pathogenic milk-borne organisms. Also, as we have previously suggested, it is from rural districts of England that some of the worst relative health returns on occasion appear, and anything which tends to bring their sanitary conditions, size for size, population for population, up to the city standards, is worthy of notice. The question of municipal milk control opens up at least one path by which the problem might be attacked, and the advisability of establishing small pasteurisation plants and these in much smaller communities than has been the recent custom, is a step which might well receive the consideration of those local health authorities concerned.

